# Weight Bias Internalization and Eating Disorder Psychopathology in Treatment-Seeking Patients with Obesity

**DOI:** 10.3390/nu15132932

**Published:** 2023-06-28

**Authors:** Simona Calugi, Barbara Segattini, Gianmatteo Cattaneo, Mirko Chimini, Anna Dalle Grave, Laura Dametti, Manuela Molgora, Riccardo Dalle Grave

**Affiliations:** Department of Eating and Weight Disorders, Villa Garda Hospital, Via Monte Baldo 89, 37016 Garda, VR, Italy; si.calugi@gmail.com (S.C.); barbara.segattini93@gmail.com (B.S.); gianmatteocattaneo@gmail.com (G.C.); mirko.chimini1@gmail.com (M.C.); annadallegrave@icloud.com (A.D.G.); lauradametti96@gmail.com (L.D.); molgoramanuela@gmail.com (M.M.)

**Keywords:** obesity, weight bias, body image, body dissatisfaction, exposure avoidance, shape dissatisfaction, empty stomach

## Abstract

This study aimed to investigate the relationship between weight bias internalization and eating disorder psychopathology in treatment-seeking patients with severe obesity using a network approach. Two thousand one hundred and thirteen patients with obesity were consecutively admitted to a specialist clinical unit for obesity and were recruited from January 2016 to February 2023. Body mass index was measured, and each patient completed the Weight Bias Internalization Scale (WBSI) and the Eating Disorder Examination Interview (EDE). Network analysis showed that the most central and highly interconnected nodes in the network were related to the EDE items exposure avoidance, dissatisfaction with shape, and wanting an empty stomach. Bridge nodes were found, but the bootstrap difference test on expected bridge influence indicated non-significant centrality differences. Nevertheless, the eating disorder psychopathology and weight bias internalization network structure in patients seeking treatment for obesity indicate the prominent roles of body dissatisfaction and control of eating and weight in these psychological constructs. This finding, if replicated, could pave the way for a new understanding of the psychological mechanisms operating in patients with obesity.

## 1. Introduction

Weight bias internalization and eating disorder psychopathology are two emerging psychological constructs with a relevant association with obesity. Weight bias refers to negative attitudes and manifested beliefs involving stereotypes, rejection, and prejudice directed toward people who are perceived as having excess body weight [1]. In contrast, internalized weight bias refers to people’s self-directed stigmatizing attitudes based on social stereotypes about their perceived weight status [2]. Weight bias internalization occurs when individuals engage in self-blame and self-directed weight stigmatization because of their weight [3]. It is also pervasive and potentially damaging to health beyond body weight and experiences of stigma [4,5]. More specifically, research has shown that a greater level of weight bias internalization predicts lower core self-evaluation, which in turn predicts greater depression and anxiety, lower global health, and greater healthcare utilization [6]. Internalized weight stigma is also significantly associated with greater eating disorder psychopathology and depression, and lower perceived mental quality of life in adult patients with loss-of-control eating after sleeve gastrectomy surgery [7]. Interestingly, a study examining the frequency of experiencing weight stigma and physiological risk factors in patients with overweight/obesity found that weight stigma was significantly linked to cortisol levels, as well as higher levels of oxidative stress, concluding that weight stigma may contribute to the poor health underlying some forms of obesity [8].

Only a few studies have evaluated the relationship between weight bias internalization and eating disorder psychopathology in patients with obesity [4]. One study, on a community sample of 228 treatment-seeking adults with overweight or obesity, found that weight bias internalization was positively associated with eating concerns, weight concerns, and shape concerns [9]. That study also tested the impact of weight bias internalization on the relationship between perceived weight discrimination and eating pathology (including binge eating, emotional eating, bulimic symptoms, and drive for thinness), revealing that weight bias internalization mediates this relationship even after controlling for body mass index (BMI). Another study collected data on two large samples of college students to assess a theoretical model designed to collectively account for the intermediary role of weight bias internalization and body dissatisfaction in associations between weight stigma experiences and a variety of eating disorder behaviours across the weight spectrum [10]. The data supported the proposed model, and, although patterns of associations differed among individuals with different BMIs, these variations were limited. Finally, two studies specifically investigated the relationship between overvaluation of shape and weight and weight bias internalization. The first, using mediation analysis, found that overvaluation of shape and weight mediates the relationship between self-esteem and weight bias internalization [11]. The second, investigating the relationship between rumination and both overvaluation of shape/weight and eating disorder psychopathology, found a significant positive association among these constructs [12].

However, the abovementioned studies used the general constructs (latent variables) of weight bias internalization and eating disorder psychopathology to investigate their relationship. Nevertheless, latent variable models of psychopathology are not without limitations [13,14]. A new theory, called network theory, suggests that psychopathology arises from a complex array of causal and reciprocal relationships among symptoms rather than directly from latent diagnoses [14,15]. The statistical approach reflecting this theory, the network analysis, has been applied to address the limitations of prior latent variable model approaches aiming to identify specific relationships between clinical features and central symptoms [14] and produce graphical and quantitative modelling of associations between constructs.

To date, no study has evaluated the complex array of reciprocal relationships among specific characteristics of the two constructs, rather than the latent constructs as a whole.

With this in mind, network analysis was used to investigate the relationship between the single characteristics of eating disorder psychopathology and weight bias internalization in more depth in a sample of treatment-seeking patients with obesity.

## 2. Materials and Methods

### 2.1. Participants

The study sample comprised 2113 patients with obesity admitted to the Villa Garda Hospital Department of Eating and Weight Disorders’ inpatient residential rehabilitative treatment programme between January 2016 and February 2023 upon completion of the baseline assessment. Eligibility criteria for this study were: age ≥ 18 years, BMI ≥ 30.0 kg/m^2^, and Comprehensive Appropriateness Scale for the Care of Obesity in Rehabilitation (CASCO-R) global score > 25. Of note, individuals with a CASCO-R score of >25 experience at least one weight loss-responsive comorbidity and several complications of obesity [16]. Exclusion criteria were pregnancy or lactation; any medications that affect body weight; severe psychiatric disorders (i.e., bulimia nervosa, substance use disorders, bipolar and related disorders, or schizophrenia spectrum/other psychotic disorders), assessed via clinical interview; and any medical comorbidity associated with weight loss.

Approval for the study was granted by the GHC Institutional Review Board (Protocol Code 0005GHCIRB). Each participant provided informed written consent for collection of their clinical data, as well as their anonymous processing in a service-level research setting.

### 2.2. Measures

All study data were collected on the second day after admission to the unit. Specifically, this involved the admitting physician filling in the following measures on a case report form for each participant:

- Weight (baseline), measured on medical weighing scales (Seca Digital Wheelchair Scale Model 664);
- Height, measured with a stadiometer (Wall-Mounted Mechanical Height Rod Model 00051A; Wunder);
- BMI, calculated using the standard formula (i.e., body weight (kg) divided by height (m) squared);
- Eating disorder features, assessing responses to the Eating Disorder Examination interview (EDE), Italian version [17]. This semi-structured questionnaire is designed to evaluate eating disorder psychopathology and behaviours in the 28 days before the interview is conducted. Specifically, scores of 0–6 are assigned to the behavioural symptoms (binge eating, self-induced vomiting, laxative misuse, diuretics misuse, excessive exercising, and food restriction) exhibited by individuals with eating disorders. EDE scores can be expressed on a global scale, but also four specific subscales (Restraint, Eating Concern, Weight Concern, and Shape Concern) reflecting the respective cognitive features. Excellent criterion validity and high test–retest reliability (r = 0.80) have been reported for the Italian version of the EDE, whose global score has very good inter-rater reliability (rho = 0.97) [17]. In our sample, Cronbach’s α for the global EDE score was 0.85. For the purposes of this study, the 22 items used to generate the four subscales and the global score were considered. The EDE was administered by assessors trained and supervised by RDG, an expert on the instrument;
- Weight bias internalization, assessed using the Italian version of the Weight Bias Internalization Scale (WBIS) [18]; this relies on a total of 11 items, rated on a seven-point Likert scale, to measure self-directed, weight-related stigma. The Cronbach’s α for the global WBIS score was 0.80 in our sample.

### 2.3. Statistical Analysis

SPSS, version 27, was used for data processing and descriptive analysis, while network analysis was conducted via R software, version 3.5.2 [19] in the RStudio environment RStudio 2023.03.0 + 386. Variables are presented either as means and standard deviations or frequencies and percentages, as appropriate. According to the Shapiro–Wilk normality test, study variables were not normally distributed, so nonparametric correlations were calculated using nonparanormal transformation [20].

#### 2.3.1. Network Estimation

The qgraph Rpackage was used to perform the network analysis [21]. Regularized partial correlation networks [22] were estimated using EBIC graphical LASSO [23]; when data points were ordinal, polychoric correlations served as input. As regularization applies an extra penalty for model complexity when estimating a statistical model, the resulting models are conservative and easier to interpret [24]; indeed, small or unstable correlations are estimated to be zero, thereby removing the connections between nodes (the network “edges”) that are less likely to be meaningful.

Fit optimization of the networks generated in this manner was carried out by minimizing the Extended Bayesian Information Criterion (EBIC) [25], an approach that is reportedly very effective at revealing the true network structure [26,27], and thereby facilitates selection of the best network; the approach functions particularly well when the generating network is sparse, containing a limited number of edges.

In order to identify nodes measuring the same underlying construct, the goldbricker function (Rpackage networktools) was used with the threshold set at 0.25; the net reduce function was applied to combine all node pairs falling below this threshold.

#### 2.3.2. Bridge Nodes

The degree to which a node in one cluster relates to nodes in another cluster can be assessed via bridge metrics. The expected influence of a bridge is the sum of the values of all edges that connect a particular node to all nodes that do not belong to the same community [28]; this approach can be used to quantify the strength and directionality of all the associations a node displays in a specific cluster. In this case, it highlighted the relationship between nodes in the eating disorder cluster (all EDE items and BMI) with nodes in the weight bias internalization cluster (all WBIS items). The node with the highest bridge centrality (see below) within each cluster was identified as the bridge node.

#### 2.3.3. Centrality Indices

The centrality indices of the network structure generated were calculated as a means of evaluating the relative importance of each of its nodes [15,22]. The expected influence (EI) of the node centrality index was then calculated and normalized (mean = 0 and standard deviation (SD) = 1). This is a means of quantifying the strength and directionality of the relationships each node has with the other nodes [22], with a value of <1 indicating that the EI is <1 SD from the mean. Expected influence is a centrality measure suggested by Robinaugh and colleagues [29] when dealing with a node’s importance in activating or deactivating other nodes in a network that has negative edges. We decided to use EI, rather than strength, to consider the directionality of the edges.

#### 2.3.4. Network Robustness, Stability, and Accuracy 

The robustness of each resulting network was estimated via calculation of the accuracy of edge weights in each network; the accuracy of the edge weights was calculated by generating nonparametric bootstrapped 95% confidence intervals (CIs) around the original edge values (n boots = 5000) [23], with narrower CIs indicating greater accuracy [23]. Pairwise bootstrapped difference testing (n boots = 5000) was then used to identify any significant differences between the two networks’ edge weights and the centrality index [22].

The EI stability was also calculated for portions of the data, following the procedure suggested by Epskamp et al. [22]. This involved random sampling of networks of nodes a thousand times, and then calculation of subset bootstraps and correlation stability (CS) coefficients. To allow interpretation of differences in centrality, the CS coefficient must be no lower than 0.25, and preferably above 0.5 [22]. This can be taken as the greatest proportion of cases that can be dropped while preserving 95% probability of the correlation between the original centrality index and the centrality of networks based on subsets being 0.7 or greater.

## 3. Results

### 3.1. Patient Characteristics

The sample comprised 2113 treatment-seeking patients with obesity, more than half of whom were female (65.4%); the mean age was 55.0 years (SD = 14.0), and the mean BMI was 41.6 kg/m^2^ (SD = 7.9).

### 3.2. Network Structure

The network analysis encompassed all raw data of the 11 WBIS items, the 22 items of the EDE interview, and the BMI, making a total of 34 items. Table 1 details each variable included in the network. The goldbricker function indicated no overlap among variables.

**Figure 1:**
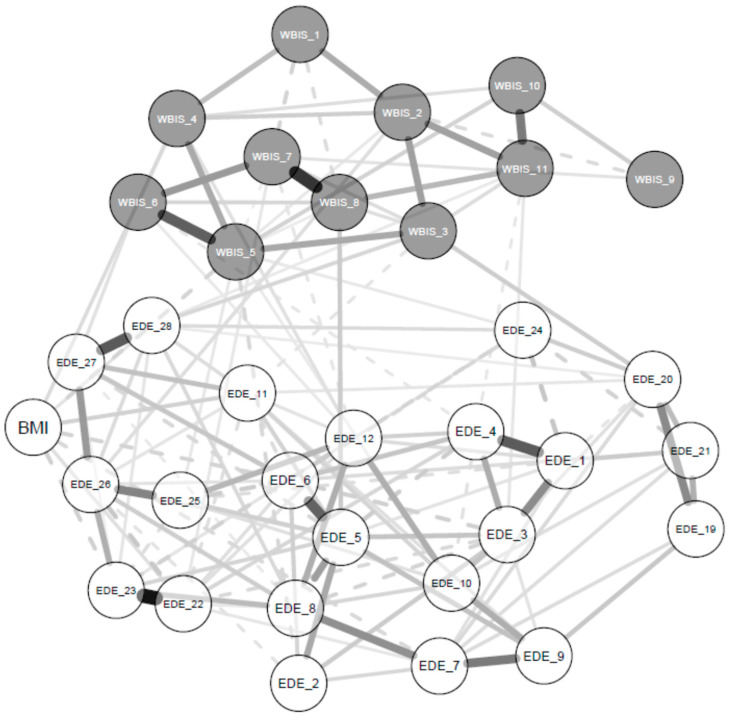
The network structure estimated from the graphical Least Absolute Shrinkage and Selection Operator in combination with Extended Bayesian Information Criterion model selection in patients with obesity. White nodes = eating disorder psychopathology; grey nodes = internalized weight bias. Solid edges = positive associations; dashed edges = negative associations; thicker edges = stronger associations. See Table 1 for items corresponding to each node.

**Figure 2:**
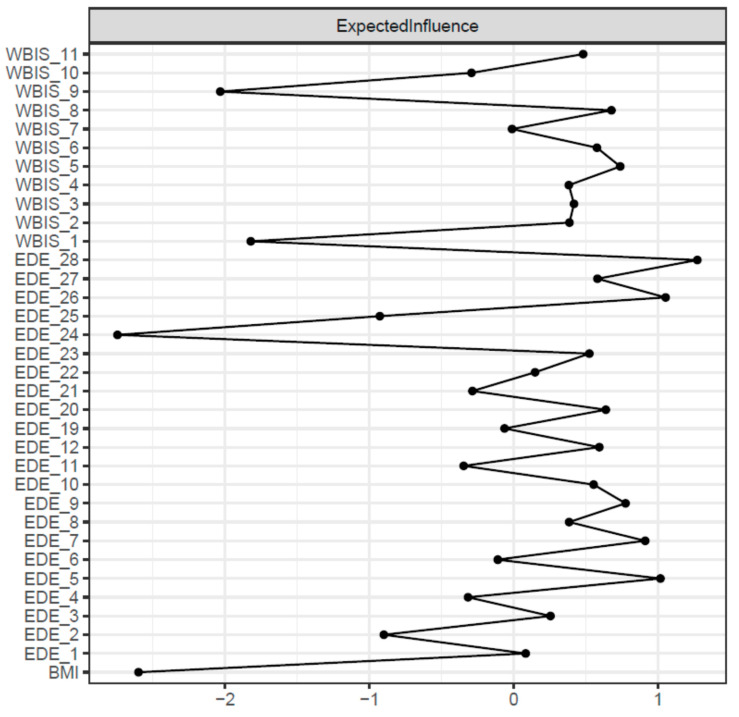
Centrality plots for adaptive Least Absolute Shrinkage and Selection Operator Network in combination with Extended Bayesian Information Criterion model selection for adult patients with obesity. Expected influence values of each node are presented. Centrality indices are shown as standardized z-scores. Abbreviations for each node are provided in Table 1.

Figure 1 and Figure 2 show the network structure and expected influence values for the global sample. Nodes presenting higher expected influence were all EDE items, specifically: ‘avoidance of exposure’ (EI = 1.27), ‘dissatisfaction with shape’ (EI = 1.04), and ‘wanting an empty stomach’ (EI = 1.00). Node strengths were stable, and the CS coefficient for EI was 0.67 (a cut-off of 0.5 is required to consider the metric stable) (Figure S1).

The bootstrapped difference test for EI values showed that EDE measures ‘avoidance of exposure’, ‘dissatisfaction with shape’, and ‘wanting an empty stomach’ had significantly greater EI centrality than other symptoms (Figure S2). The bootstrapped 95% CIs around the estimated edge weights indicated that many of the edge weights did not significantly differ from one another (Figure S3).

Analysis of the bridge nodes indicated that in the specific eating disorder psychopathology cluster, the greatest bridge EI corresponded to the EDE measure ‘avoidance of exposure’. In the weight bias internalization cluster, on the other hand, the greatest bridge EI corresponded with the WBIS measure ‘I wish I could drastically change my weight’ (Figure S4). However, the bootstrap difference test on the bridge EIs indicated non-significant centrality differences, thereby failing to confirm the role of the two nodes as bridge nodes (Figure S5).

## 4. Discussion

This study used a network approach to assess the relationships between weight bias internalization and eating disorder psychopathology in a large sample of treatment-seeking patients with obesity. There were two main findings.

Upon inspection of the network, the first finding was that certain symptoms, namely exposure avoidance, shape dissatisfaction, and wanting an empty stomach, all in the eating disorder psychopathology cluster, were central nodes with strong connections to all the other eating disorder and weight stigma variables in the network. Avoidance of exposure and dissatisfaction with shape, two items on the EDE shape concern subscale, represent cognitive and behavioural features of body image dissatisfaction. Several studies have suggested a positive relationship between body image dissatisfaction and obesity [30,31,32], and a meta-analysis of 17 quantitative studies on adult samples found that individuals with obesity reported greater body dissatisfaction than normal weight individuals [33]. However, the specific features of body image dissatisfaction in patients with obesity individuated in this study may shed light on the most influential psychological mechanism related to eating disorder psychopathology and weight bias internalization; these features could, more so than others, provide the motivation to seek treatment in patients with obesity. In addition, the identification of the desire to have an empty stomach as a central node in the network reflects the relevant role of this feature in patients with obesity. This feature, measured via the EDE restraint subscale, is similarly influential in underweight patients with an eating disorder [34,35], indicating that it could be an important factor in eating disorder psychopathology and weight bias internalization due to its interpretation in terms of control over eating and weight, at least in treatment-seeking patients with obesity.

The second finding from our analysis was that the bridges nodes linking the two separate constructs are not stable, with non-significant centrality differences among nodes. Explaining this finding is complex. Nevertheless, we hypothesize that the two constructs (eating disorder psychopathology and weight bias internalization) were made up of very similar variables, with comparable relationship strengths between them, and that the two latent variables measured are only artificially constructed in patients with obesity. However, it could also signify that all nodes are potentially relevant as bridges between eating disorder psychopathology and weight bias internalization.

The study had three main strengths. First, to our knowledge it is the first to use a network approach to investigate the interconnections between eating disorder psychopathology and weight bias internalization in a large sample of patients with obesity. Second, the very large sample permits conclusions about the population of treatment-seeking patients with obesity to be drawn. Third, the use of the EDE interview, conducted by a clinician expert in eating disorders and obesity to assess eating disorder psychopathology provides confidence in the accuracy and reliability of evaluation of these features.

However, the study does present some limitations. The first is that all patients were seeking treatment, and therefore no inference can be drawn regarding the general population of individuals with obesity. Another limitation is the cross-sectional nature of the study, which prevents inferences about the directionality of the relationships we detected to be made. This means that we are not in a position to draw conclusions about clinical treatment, and we can merely point toward avenues for future research.

## 5. Conclusions

In conclusion, our data indicate that body dissatisfaction and control of eating and weight had a prominent role in the eating disorder psychopathology and weight bias internalization network structure in patients seeking treatment for obesity. Overall, these findings represent the first step in a new way to understand the psychological mechanisms operating in patients with obesity.

## Figures and Tables

**Table 1:** Variable abbreviations in node network of 34 items.

BMI	Body Mass Index
EDE_1	Restraint over eating
EDE_2	Avoidance of eating
EDE_3	Food avoidance
EDE_4	Dietary rules
EDE_5	Wanting an empty stomach
EDE_6	Flat stomach
EDE_7	Preoccupation with food, eating or calories
EDE_8	Preoccupation with shape or weight
EDE_9	Fear of losing control over eating
EDE_10	Fear of weight gain
EDE_11	Feelings of fatness
EDE_12	Desire to lose weight
EDE_19	Eating in secret
EDE_20	Social eating
EDE_21	Guilt about eating
EDE_22	Importance of weight
EDE_23	Importance of shape
EDE_24	Reaction to prescribed weighing
EDE_25	Dissatisfaction with weight
EDE_26	Dissatisfaction with shape
EDE_27	Discomfort seeing body
EDE_28	Avoidance of exposure
WBIS_1	As an overweight person, I feel that I am just as competent as anyone
WBIS_2	I am less attractive than most other people because of my weight
WBIS_3	I feel anxious about being overweight because of what people might think of me
WBIS_4	I wish I could drastically change my weight
WBIS_5	Whenever I think a lot about being overweight, I feel depressed
WBIS_6	I hate myself for being overweight
WBIS_7	My weight is a major way that I judge my value as a person
WBIS_8	I don’t feel that I deserve to have a really fulfilling social life, as long as I’m overweight
WBIS_9	I am OK being the weight that I am
WBIS_10	Because I’m overweight, I don’t feel like my true self
WBIS_11	Because of my weight, I don’t understand how anyone attractive would want to date me

## Data Availability

The data that support the findings of this study are available from the corresponding author, SC, upon reasonable request.

## Supplementary Materials

The following supporting information can be downloaded at: https://www.mdpi.com/article/10.3390/nu15132932/s1, Figure S1: Average correlations between centrality indices of networks sampled with persons dropped and the original sample. Lines indicate the means, and areas indicate the range from the 2.5th quantile to the 97.5th quantile. Figure S2: Bootstrapped difference tests (α = 0.05) for centrality within the network. Nodes are presented in descending order of centrality. Values on the diagonal indicate the unstandardized centrality estimates for each node. Black boxes indicate significant centrality differences, meaning that the bootstrapped difference 95% CI does not span 0. Grey boxes indicate non-significant centrality differences, meaning that the bootstrapped difference 95% CI spans 0. See Table 1 for items corresponding to each node. Figure S3: Bootstrapped 95% confidence intervals (CIs) of estimated edge weights for the network. The red line indicates the sample values, and the grey area the bootstrapped 95% CIs. Each horizontal line represents one edge of the network, ordered from the edge with the highest weight to that with the lowest. The y-axis labels have been removed to avoid cluttering. Figure S4: Bridge expected influence among the eating disorder psychopathology and weight bias internalization symptoms network. See Table 1 for items corresponding to each node. Figure S5: Bootstrapped difference tests (a = 0.05) for expected influence of bridges within the network. Nodes are presented in descending order of centrality. Black boxes indicate significant centrality differences, meaning that the bootstrapped difference 95% CI does not span 0. Grey boxes indicate non-significant centrality differences, meaning that the bootstrapped difference 95% CI spans 0. See Table 1 for items corresponding to each node.
