# Supplementary figures and images for: Weight Bias Internalization and Eating Disorder Psychopathology in Treatment-Seeking Patients with Obesity
Source: Nutrients. 2023 Jun 28;15(13):2932. doi: 10.3390/nu15132932 (PMC10346744; doi:10.3390/nu15132932)

Average correlation with original sample

expectedInfluence

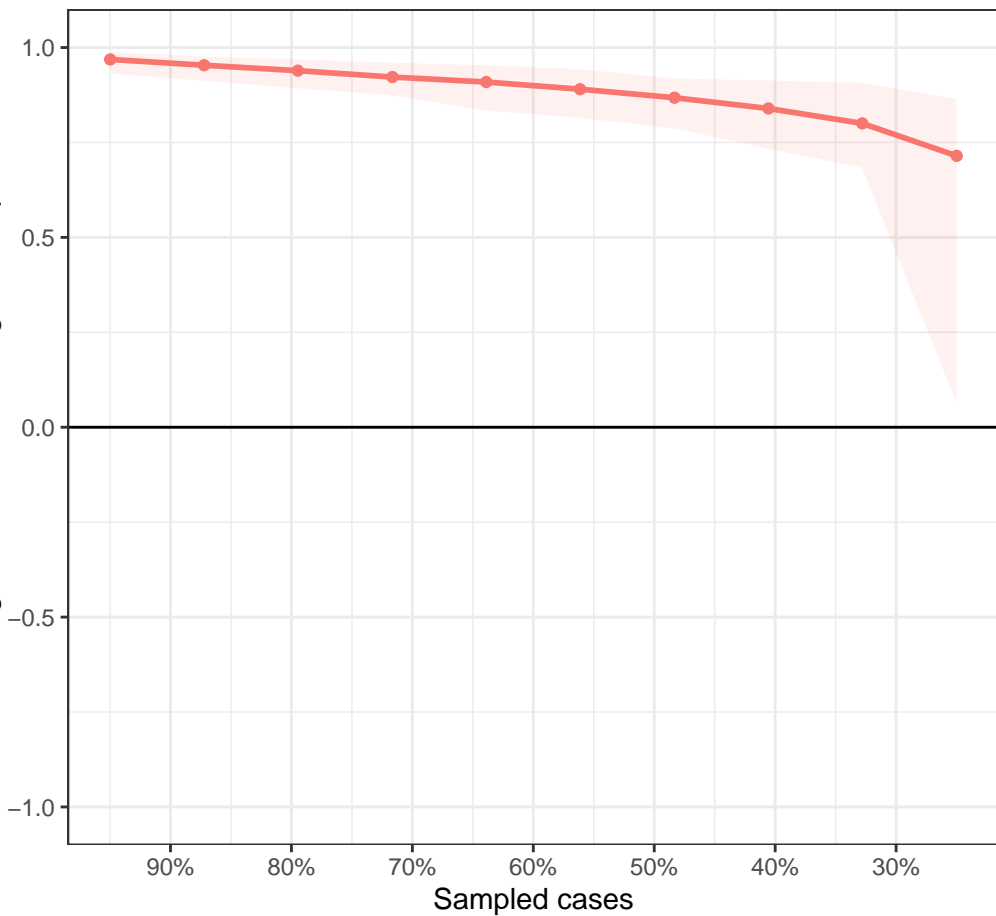

Supplement: Supplementary file 1 [file nutrients-15-02932-s001.zip › Figure S1.pdf]

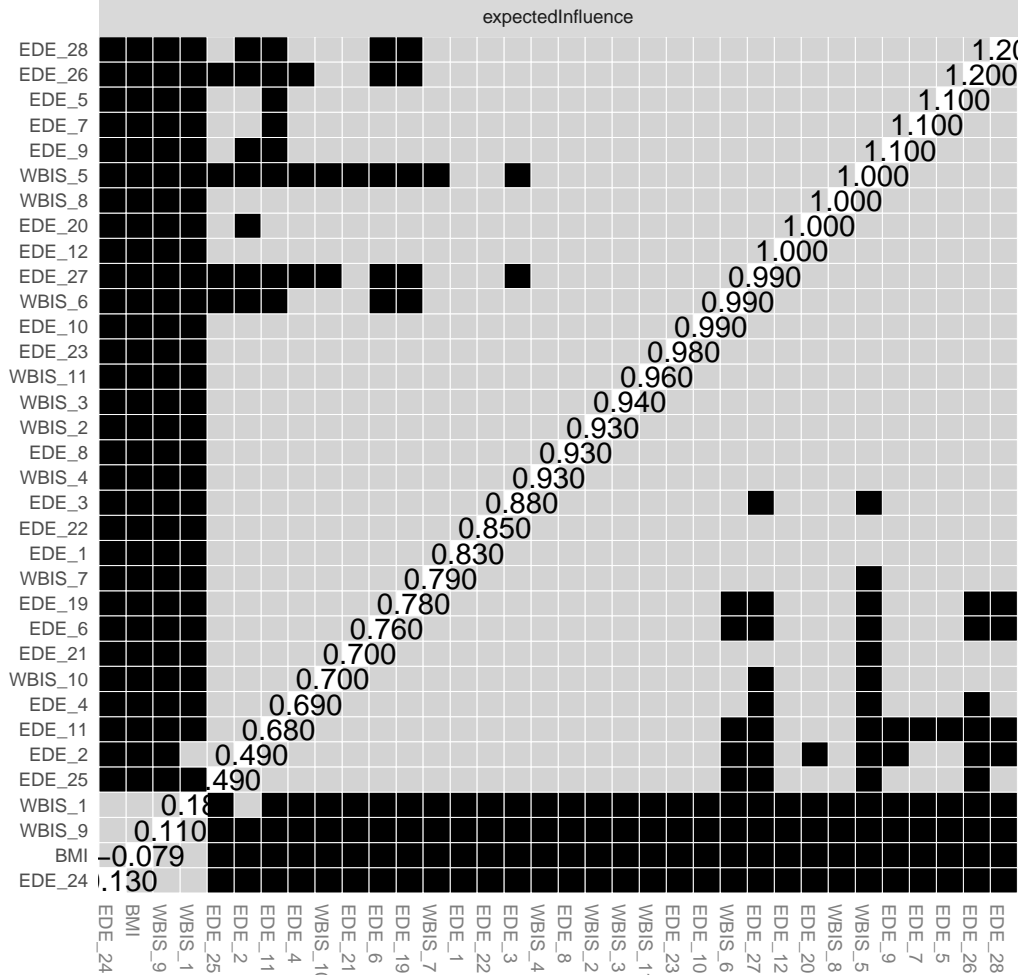

Supplement: Supplementary file 1 [file nutrients-15-02932-s001.zip › Figure S2.pdf]

expectedInfluence

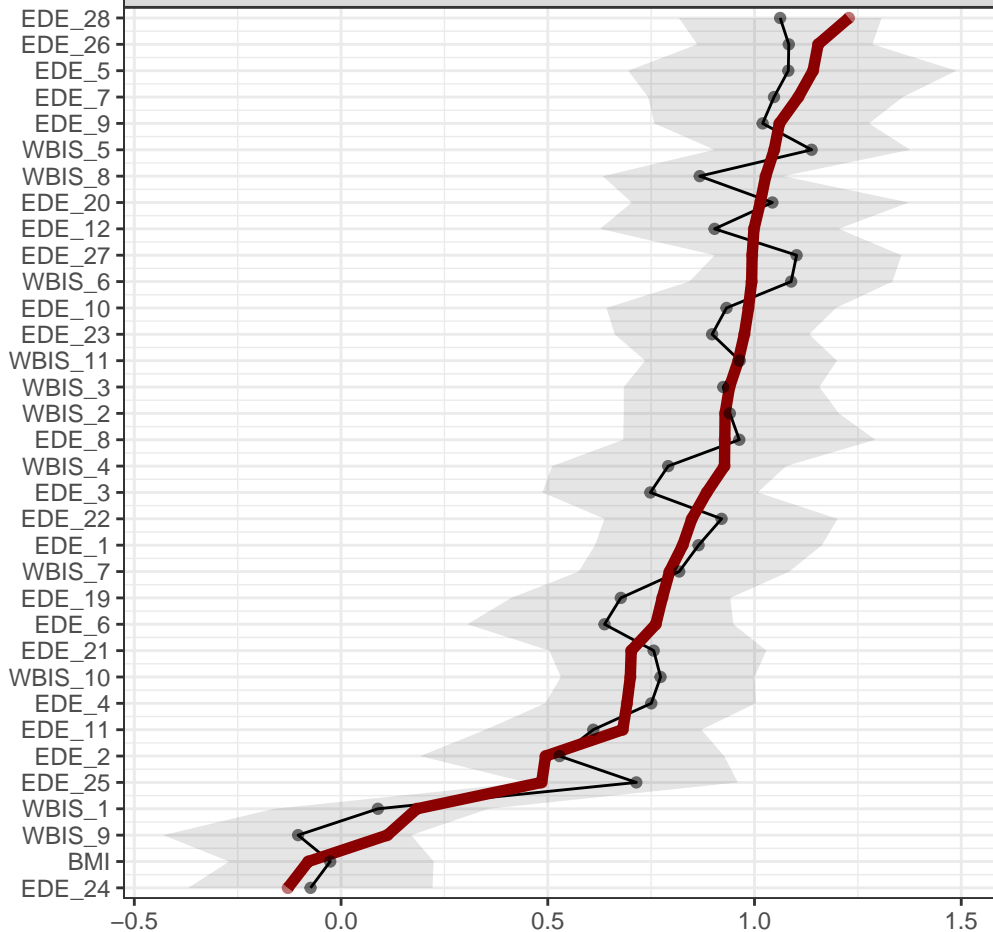

Supplement: Supplementary file 1 [file nutrients-15-02932-s001.zip › Figure S3.pdf]

Bridge Expected Influence (1-step)

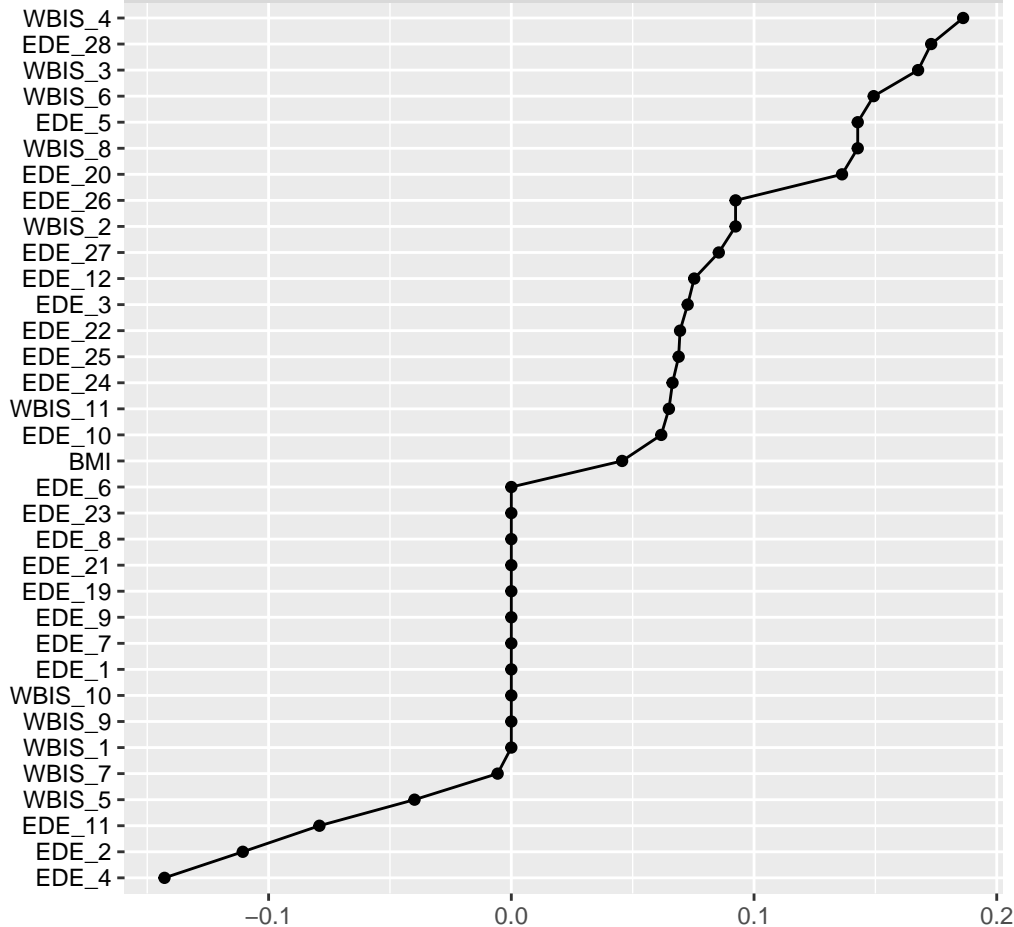

Supplement: Supplementary file 1 [file nutrients-15-02932-s001.zip › Figure S4.pdf]

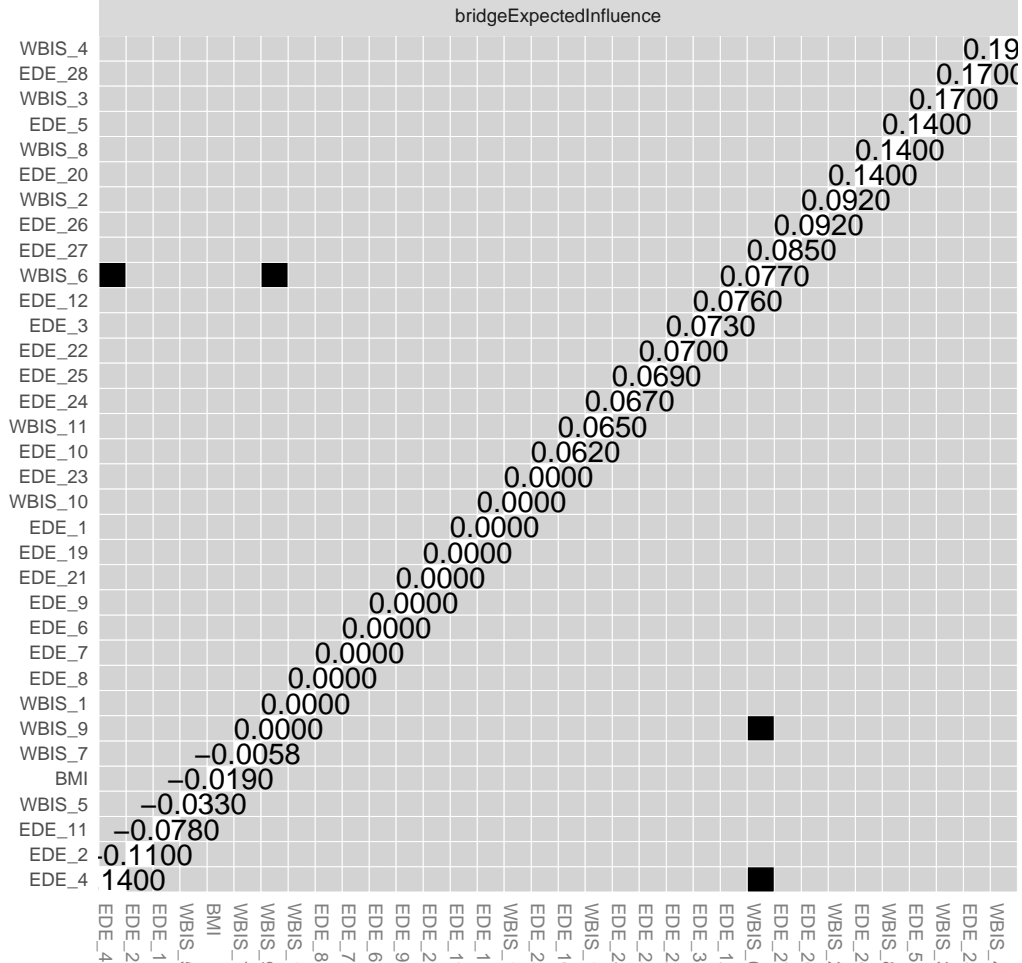

Supplement: Supplementary file 1 [file nutrients-15-02932-s001.zip › Figure S5.pdf]
